# Development of an Empirical Approach for the Prediction of Cytochrome P450-Based Drug–Drug Interactions in Pediatric Patients

**DOI:** 10.3390/ph19040608

**Published:** 2026-04-10

**Authors:** Veronica Di Paolo, Francesco Maria Ferrari, Italo Poggesi, Luigi Quintieri

**Affiliations:** 1 Laboratory of Drug Metabolism, Department of Pharmaceutical and Pharmacological Sciences, University of Padua, 35131 Padua, Italy; 2 Department R&D, MeteRSit, 35129 Padua, Italy; fraul189@gmail.com; 3 Quantitative Clinical Pharmacology, GSK, 37135 Verona, Italy; italo.x.poggesi@gsk.com

**Keywords:** prediction, drug–drug interactions, pediatrics, static method

## Abstract

**Background and Objective**: Predicting drug–drug interactions (DDIs) in pediatric patients remains a major challenge in clinical pharmacology. This study aimed to evaluate and compare three empirical approaches for extrapolating adult cytochrome P450 (CYP)-mediated DDI pharmacokinetics (PK) data to predict the extent of the corresponding DDIs in children across different age groups. **Methods**: The approaches assessed were: (A) the direct use of adult area under the plasma concentration–time curve ratios (AUCRs) as estimators of pediatric values; (B) the application of a correction accounting for the ontogeny of the involved CYP enzyme; and (C) the application of corrections for both enzyme ontogeny and allometric scaling. Twenty-five pediatric AUCRs were predicted from adult AUCR data. Predictive performance was evaluated by comparing predicted AUCR_pediatric_ values with observed values, using a 50–200% acceptability range. **Results**: Approach C demonstrated superior predictive capability, with only one out of 25 predictions falling outside the acceptability range. In contrast, both approaches A and B resulted in three values each outside this range. Further visual exploration and detailed performance analyses confirmed the enhanced accuracy of approach C in predicting pediatric DDIs compared with the other approaches. **Conclusions**: This study demonstrates that the proposed approach of considering both ontogeny and allometric scaling represents a robust and reasonable method to anticipate the extent of pediatric CYP-based DDIs when adult PK data are available.

## 1. Introduction

The practice of polypharmacy has become increasingly widespread, not only in adults but also in children, leading to a high risk of potentially harmful pediatric drug interactions (DDIs) [1]. It is noteworthy that severe DDIs that may require medical intervention or be life-threatening occur in 41% of pediatric hospital admissions [2], and that the extent of a DDI in children may differ from that in adults [3] due to age-related physiological changes that can significantly alter a drug’s pharmacokinetics (PK) and/or pharmacodynamics [4]. In particular, it is well recognized that substantial changes in PK parameters occur during human development [5], and these changes contribute to the differences in therapeutic efficacy and adverse drug events observed in children compared to adults [6].

One of the main problems in pediatric pharmacotherapy is the poor availability of PK data from clinical studies conducted on children relative to adults, and both ethical concerns and regulatory restrictions limit the ability to perform comprehensive clinical studies, including DDI studies, in pediatric populations [3,7]. Consequently, the development and utilization of prediction systems for DDIs in pediatrics have become increasingly popular. In the absence of specific clinical evidence, the default approach is to extrapolate DDIs from adults to pediatric individuals [8]. For over two decades, the technique of allometric scaling (AS) has been employed to translate adult PK data to pediatric settings, and numerous publications have extensively explored its underlying mechanisms, with particular attention to its theoretical foundations [9,10,11]. While DDI studies conducted on adult volunteers provide valuable information, they should be considered merely an initial step in understanding potential DDIs in pediatrics. In particular, it is crucial to be aware that results based on studies conducted in adults may not fully capture the clinical relevance of DDIs across the various stages of child development [8].

In recent years, there has been an increasing use of physiology-based pharmacokinetics (PBPK) models to predict the fate of a drug in the body through a system of differential mass balance equations that describe the processes of absorption, distribution, metabolism, and excretion (ADME) [12]. Although these systems may facilitate the assessment of potential PK-based DDIs in pediatrics, there are many knowledge gaps regarding age-related changes in ADME in pediatric populations [7].

An important aspect influencing the potential for a DDI in pediatric subjects, compared to adults, is the variability in drug clearance associated with the ontogeny of drug-metabolizing enzymes (DMEs), in particular the primary phase I enzymes involved in drug metabolism, i.e., cytochrome P450s (CYPs) [13,14]. According to their ontogenic expression pattern, DMEs can be divided into three classes [13,14,15]:

- Class 1 DMEs, exemplified by CYP3A7 [16], are expressed at their highest levels in the fetus during the first trimester, and their levels either remain elevated or gradually decline during gestation. These enzymes are silenced or expressed at relatively low levels within a few days to as long 1–2 years *postpartum*.
- Class 2 DMEs, exemplified by CYP2B6, CYP2C19 and CYP3A5 [16,17,18], display relatively stable expression throughout gestation and into adulthood. Expression of these proteins may increase moderately (as in the case of CYP2C19) or not within the first year after birth.
- Finally, DMEs belonging to class 3, among which are CYP2C9, CYP2D6, and CYP3A4 [16,18,19], are not expressed or expressed at negligible levels in the fetus. For many of these enzymes, the onset of expression is observed in the second or third trimester of gestation, but levels remain substantially lower than those observed at full maturation. Substantial increases in expression to mature levels typically occur within the first 12–24 months *postpartum*.

It is noteworthy that the majority of both phase I and II DMEs belong to class 3, and that the perinatal period represents a time window of considerable interindividual variability in expression for most of both class 1 and class 3 enzymes, although the duration of this time frame varies considerably from one DME to another [14]. Changes in the expression of DMEs that occur during development can significantly impact the efficacy and toxicity of a drug, as well as influence the risk of DDIs in infants and children [20]. In particular, in the neonate and young child, inhibition of the major metabolic pathway of a given drug can have profound PK consequences if an alternative elimination pathway (or pathways) has not adequately matured. Indeed, under such circumstances, the affected drug may accumulate, resulting in a DDI of greater magnitude than that observed in adults [8].

The magnitude of a PK DDI can be quantified using the AUC ratio (AUCR). AUCR is defined as the ratio between the AUC of a victim drug (a CYP substrate) administered in combination with a DDI perpetrator (i.e., an inhibitor or inducer of the major CYP involved in the victim drug’s metabolism) and the AUC of the victim drug when administered alone. Static approaches are available (e.g., [21,22]) and have been successfully applied to anticipate the expected AUCR in adults.

In this work, empirical corrections—accounting for either the ontogenetic expression of the relevant CYP enzyme, the age-dependent body weight of pediatric patients, or both—were evaluated to predict the pediatric AUCR from those obtained in adults.

## 2. Results

**Figure 1:**
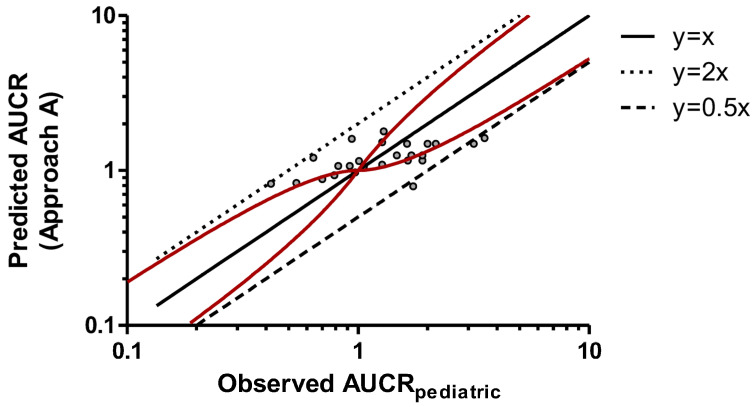
Predicted AUCR (Approach A) versus observed AUCR_pediatric_. Solid red curves denote intervals, as suggested by Guest et al. [23]. The black solid line is the identity line (y = x). The upper and lower dashed lines represent y = 2x and y = 0.5x, respectively. Linear regression analysis led to the following equation: y = 0.2052x + 0.9259. AUCR: area under the plasma concentration–time curve ratio.

**Figure 2:**
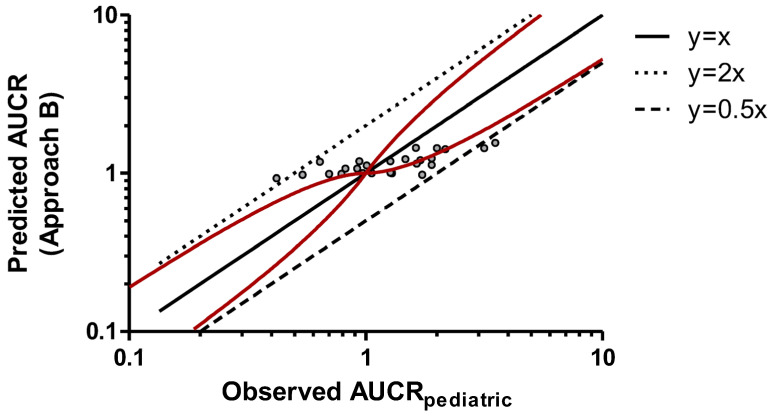
Predicted AUCR (Approach B) versus observed AUCR_pediatric_. Solid red curves denote intervals, as suggested by Guest et al. [23]. The black solid line is the identity line (y = x). The upper and lower dashed lines represent y = 2x and y = 0.5x, respectively. Linear regression analysis led to the following equation: y = 0.1909x + 0.8866. AUCR: area under the plasma concentration–time curve ratio.

In this study, three empirical approaches for predicting CYP-based DDIs in pediatric patients were explored. Approach A involved a direct translation of the observed AUCR_adult_ values to pediatric patients, assuming that they were equal. The relationship between observed AUCR_adult_ and AUCR_pediatric_ values was examined and is plotted in Figure 1. Approach B, illustrated in Figure 2, considered only an ontogeny correction factor (variable Z_ontogeny_) without applying an allometric correction factor (variable W_allometry_). Finally, Approach C extrapolated adult PK data to pediatric patients, incorporating both ontogeny and allometric scaling factors. The graphical representation of predicted *versus* observed AUCR values in pediatric subjects enabled a side-by-side evaluation of the three approaches, offering insights into their relative performance and potential limitations. The AUCR values predicted with the three methods are reported in Table 1.

**Figure 3:**
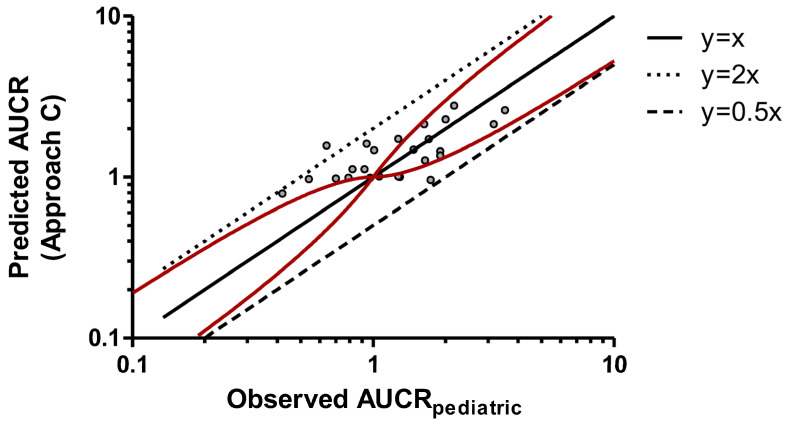
Predicted AUCR (Approach C) versus observed AUCR_pediatric_. The red solid curves denote the intervals, as suggested by Guest et al. [23]. Solid black line is the identity line (y = x). The upper and lower dashed lines represent y = 2x and y = 0.5x, respectively. Linear regression analysis led to the following equation: y = 0.5382x + 0.6978. AUCR: area under the plasma concentration–time curve ratio.

Among the three approaches, the third demonstrated the best predictive capability: most predicted AUCR_pediatric_ values were within 50–200% of the corresponding observed values, with only one of the twenty-five estimates exceeding the accepted upper limit (Figure 3). In contrast, both the first (direct adult-to-pediatric translation) and the second (use of ontogeny factor only) approaches yielded three values outside the acceptability range (Figure 1 and Figure 2). An analogous conclusion can be drawn from the assessment of the number of cases falling outside the Guest limits (13/25, 15/25, and 10/25 for approaches A, B, and C, respectively). Regression analysis indicated that predicted and observed AUCR values had a slope that was closer to unity for Approach C (0.5382) compared with the other approaches (0.2052 and 0.1909 for Approaches A and B, respectively), further supporting the superior predictive performance of the approach that incorporated both ontogeny and allometric corrections.

**Figure 4:**
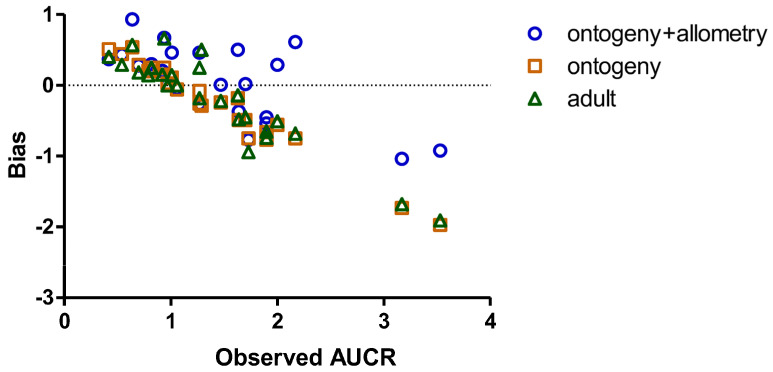
Bias of the three correction approaches as a function of the observed AUCR.

A detailed performance analysis of the three models is summarized in Table 2, providing a comprehensive comparison of their predictive capabilities and confirming the superior accuracy of the third method in predicting CYP-based pediatric DDIs, using the predicted/observed AUCR. Figure 4 shows the relationship between the absolute bias (the difference between predicted and observed AUCR) and the observed AUCR. To further characterize the predictive performance of the three approaches, the mean absolute prediction error (MAPE) and the geometric mean fold error (GMFE) were calculated. The GMFE, which is considered the most appropriate metric for PK prediction data given their inherently asymmetric distribution, confirms the superior performance of Approach C (GMFE = 1.36) compared to Approaches A (GMFE = 1.41) and B (GMFE = 1.42). MAPE values were broadly similar across the three approaches (Approach A: 34.23%; Approach B: 34.91%; Approach C: 35.61%).

## 3. Discussion

The present study evaluated three distinct approaches for predicting CYP-based pediatric DDIs, with the goal of identifying the most reliable strategy for extrapolating adult PK data to children. The empirical model integrating both ontogeny and allometric scaling demonstrated superior performance in predicting AUCRs in pediatric subjects, accurately estimating values for 24 of 25 victim–perpetrator drug combinations. Its predictive accuracy is supported by the finding that 96% of estimates fell within 50–200% of observed AUCR_pediatric_ values, a range that is commonly considered acceptable in PK predictions [24,25,26,27].

Figure 3, which visually represents values predicted with this approach *versus* observed AUCR_pediatric_ values, offers a clear illustration of the model’s performance, suggesting that this empirical approach has a better ability to predict pediatric DDIs than the other two prediction systems. The clustering of data points around the line of unity suggests a strong consistency between predicted and observed values, further validating the methodology. The conservative nature of Approach C is illustrated by the carbamazepine–erythromycin DDI, where predicted AUCR values of 2.78 and 2.29 for 6- and 8-year-old children exceeded the observed values of 2.17 and 2.00, respectively, while Approaches A and B underestimated the interaction with AUCR values below 1.5. In the context of pediatric DDI risk assessment, this tendency toward worst-case estimates may be regarded as a clinically favorable feature, as overprediction of interaction magnitude encourages appropriate precautionary measures, whereas underestimation could lead to potentially harmful drug combinations going unrecognized.

Despite the inherent challenges in pediatric PK predictions, the approach based on the correction for both ontogeny and allometry provides a remarkable predictive capacity across a wide age range, from 2 months to 18 years of age.

The digoxin–carvedilol interaction is a good example for describing the model’s capabilities. With a prediction error of 24% in a population that includes neonates as young as two weeks-old, this result is particularly encouraging. Considering the complex PK of digoxin and the significant physiological changes occurring in the neonatal period, achieving this level of accuracy is a substantial accomplishment. A similar example concerns the prediction of the etoposide–cyclosporine A interaction, which, based on the results of two different studies spanning an age range from 8 months to 20.7 years, provided prediction errors of less than 1 and 28%, better than the other explored approaches.

Of note, the correction for both ontogeny and allometry represented the most conservative estimate (i.e., it provided the worst-case scenario for the DDI) compared to the other examined approaches, especially in cases of large AUCRs (e.g., see AUCR values > 1.5 in Figure 4) following the co-administration of a substrate with strong inhibitors, which can be considered an advantage when minimizing the risk of adverse reactions in the pediatric population is a priority.

While these results are encouraging, it is important to acknowledge the limitations of this approach. We describe here three different corrections for CR and, based on our data-driven approach, the correction for both ontogeny and allometry demonstrated greater accuracy than the other methods. In particular, the proposed approach outperforms the method based on the direct translation to pediatric subjects of the AUCR value obtained in adults, which is already criticized in the literature [3]. These corrections still represent an empirical approach, which is inevitably dependent on the analyzed dataset. Despite the sample size of 25 victim–perpetrator drug combinations being relatively large, especially considering the paucity of DDI data in pediatric subjects, it would be beneficial to apply the proposed approach to a larger dataset to further confirm its accuracy. In addition, as indicated elsewhere, the adopted corrections are only applied to the parameter CR, while it is assumed that the parameters IR and IC in pediatric subjects are fixed to the adult values, which may also represent a root cause of inaccuracy if this assumption is violated. The assumption of age-invariant IR and IC values represents an inherent simplification of the proposed framework. While these parameters primarily reflect intrinsic molecular properties of the perpetrator drug, age-dependent physiological changes such as developmental alterations in hepatic blood flow, membrane composition, and intracellular environment could potentially modulate the effective *in vivo* inhibition or induction potency in pediatric subjects. Future work incorporating age-dependent IR and IC estimates, for instance derived from physiological considerations, could further refine the predictive accuracy of empirical DDI extrapolation approaches, provided that the necessary pediatric data become available.

A crucial point to consider is the inherent challenge in predicting PK in the pediatric population due to the nature of the data used in the model. For ethical reasons, pediatric DDI data are mostly obtained in pediatric patients rather than healthy subjects, whilst in most cases, DDIs are evaluated in healthy adult subjects. This discrepancy introduces an additional, unavoidable source of inaccuracies when we base our approaches based on adult data. The current framework does not account for disease-specific alterations in clearance or distribution in pediatric subjects, and such factors may contribute to residual bias in the predictions. Indeed, some of the larger prediction errors observed in our analysis, such as those involving theophylline and lidocaine DDIs, may at least partly reflect underlying disease-related changes in PK that are not captured by the empirical corrections applied. The physiological differences between patients and healthy subjects, such as altered organ function, comorbidities, or concomitant medications, may influence drug metabolism and DDIs in ways that this approach cannot capture. This limitation underscores the complexity of pediatric pharmacology and the need for cautious interpretation of the results. It should also be noted that this approach was implemented using the mean age of the pediatric assessment as the reference value for each study cohort. The paucity of individual pediatric PK data in the public domain prevented the use of the approach of more granular age stratification. Due to the limited sample size, a stratified analysis was not possible for the individual metabolic pathways; however, when focusing on CYP3A4 substrates, which represented the largest subgroup in our dataset (*n* = 14), a trend toward improved predictive performance was observed with Approach C, showing the lowest MAPE among the three approaches for CYP3A4 substrates (Approach C: 32.33%; Approach B: 40.76%; Approach A: 39.69%). Furthermore, when considering each DDI individually, Approach C showed the lowest MAPE in 9 out of 14 CYP3A4 substrates. This finding may be mechanistically explained by the prominent role of body-size-dependent scaling for this enzyme: CYP3A4 is the most abundant CYP enzyme in the human liver, and its activity may be most influenced by allometric scaling.

While the proposed empirical framework demonstrated promising predictive performance on the dataset compiled by Salem et al. [3], future studies should aim to validate these approaches against a larger number of pediatric DDI cases, ideally drawn from independent clinical datasets, to further confirm their external validity and broader applicability.

It is important to acknowledge that this empirical approach does not aim to replace PBPK modeling, which undoubtedly provides more comprehensive and mechanistic insights into PK-based DDI prediction in pediatrics. Rather, our approach serves as an empirical, pragmatic alternative when time constraints or limited resources preclude the development of a PBPK model, offering clinically useful predictions that are easy to implement. The approach proposed here can be used for anticipating the potential risk; however, confirmation with a dynamic PBPK model would always be advised [28].

These results, taken together, demonstrate that the proposed approach of correcting the equations proposed by Ohno et al. [21,22] with ontogeny and allometric factors represents a reasonable method to anticipate the extent of CYP-based pediatric DDIs when AUCR data are available in adults. By predicting DDIs across different age groups and drug combinations with reasonable accuracy, the approach addresses a critical need in pediatrics. It offers a system for obtaining an initial estimate of potential DDIs in the pediatric population in a simplified, readily applicable manner, without the need for sophisticated software.

## 4. Materials and Methods

### 4.1. Model Description

In this work, we propose different empirical methodologies to predict the AUCR in pediatric subjects.

An approach for estimating the AUCR for the oral co-administration of a CYP3A4 substrate and a CYP3A4 inhibitor in adults was described by Ohno et al. [21], who derived Equation (1) below:

(1) $$\frac{{AUC}^{*}}{AUC}=\frac{1}{1-CR*IR}$$

where $\frac{{AUC}^{*}}{AUC}$ is the ratio of the area under the plasma concentration–time curve (AUC) of the substrate drug that is co-administered with a CYP3A4 inhibitor (AUC*) to the AUC of the substrate drug given alone; CR is the fraction of the substrate’s oral clearance due to metabolism via CYP3A4; and IR is the inhibition ratio for the inhibitor and represents the *in vivo* potency of the inhibitor integrated over time.

In their seminal paper, Ohno et al. [21] also demonstrated that ${CR}_{CYP3A4}$ can be described by the following (Equation (2)):

(2) $${CR}_{CYP3A4}ADULTS=\frac{{CL}_{int(CYP3A4)}}{{CL}_{int(H)}}$$

The approach proposed by Ohno et al. [21] has subsequently been successfully applied by other research groups, including ours, for the prediction of DDIs involving other CYPs and more complex cases in both humans and veterinary species [24,25,26,29,30,31].

Within our translational framework, we explored three alternative strategies for extrapolating ${CR}_{CYP}ADULT$ to ${CR}_{CYP}PEDIATRIC$: (A) assuming equivalence between adult and pediatric values; (B) applying an ontogeny-based correction to the specific metabolic pathway; and (C) combining ontogeny adjustment with an allometric correction for body size.

Approach A—${CR}_{CYP}ADULT$ and ${CR}_{CYP}PEDIATRIC\;$ are assumed to be equivalent. Although Salem et al. [3] have suggested that data in adults may provide a biased assessment of the situation in pediatric subjects, we considered this the easiest option and the primary benchmark, serving as our first approximation.

Approach B—${CR}_{CYP}ADULT$ is corrected for ontogeny to derive ${CR}_{CYP}PEDIATRIC$. An ontogeny correction can be easily devised for the involved metabolic pathway in the numerator of Equation (2). However, an analogous correction cannot be easily developed for the overall CL_int(H)_ in the denominator of the same equation, which represents a mixture of different metabolic pathways, dependent on the characteristics of the substrate drug. Consequently, the denominator of Equation (2) remains uncorrected. The relative ontogeny of CYPs from birth onwards can be expressed via the factor *Z_ontogeny_*:

(3) $${CR}_{CYP}PEDIATRIC=\frac{Z_{ontogeny}\cdot {CL}_{int(CYP)}}{{CL}_{int(H)}}$$

which can be estimated according to the Equations published by Salem et al. [8]:

(4) $$\mathrm{CYP}1A2=\frac{1.05-0.08*{Age}^{1.1}}{1.69^{1.1}+{Age}^{1.1}}+0.08$$

(5) $$\mathrm{CYP}2B6=\frac{1-0.1*Age}{1+Age}+0.1$$

(6) $$\mathrm{CYP}2C9=\frac{1-0.17*{Age}^{0.53}}{0.016^{0.53}+{Age}^{0.53}}+0.17$$

(7) $$\mathrm{CYP}2C19=\frac{1-0.3*{Age}^{2.44}}{0.28^{2.44}+{Age}^{2.44}}+0.3$$

(8) $$\mathrm{CYP}3A4/5=\frac{1.061*{Age}^{0.78}}{0.66^{0.78}+{Age}^{0.78}}$$

Approach C—${CR}_{CYP}ADULT$ is corrected for both ontogeny (as in Approach B) and body size, via allometric scaling [10], to obtain ${CR}_{CYP}PEDIATRIC$. It should be noted that if the allometric correction for body size ${\left(\frac{{BW}_{pediatric}}{{BW}_{adult}}\right)}^{0.75}$ is applied to both the specific clearance pathway (numerator of Equations (2) and (3)) and to the total clearance describing the overall pathways (i.e., denominator of Equations (2) and (3)), this contribution would ultimately cancel out, providing a correction identical to that obtained in Approach B.

Therefore, in this approach, the correction for body size is applied only to the overall metabolic pathways (denominator of Equation (2)). The allometric correction may be described by the factor W_allometry_ in Equation (9):

(9) $${CR}_{CYP}PEDIATRIC=\frac{Z_{ontogeny}{CL}_{int(CYP)}}{W_{allometry}{CL}_{int(H)}},$$

where

(10) $$W_{allometry}={\left(\frac{{WT}_{pediatrics}}{{WT}_{adults}}\right)}^{0.75}$$

The *WT_pediatrics_* can be calculated using the formula published by Luscombe [32] (Equation (9)):

(11) $${WT}_{pediatrics}=3(\mathrm{Age})+7$$

In the absence of correction factors to apply to the IR in Equation (1), it has been assumed that the IR of an inhibitor is the same for both adults and children. It is worth emphasizing that inhibitory potency mainly reflects the intrinsic characteristics of the inhibitory drug (e.g., its affinity for the binding site of the enzyme).

Rearranging Equation (1) with the potential correction factors Z_ontogeny_ and W_allometry_, we can express the AUCR in pediatric subjects as a function of AUCR in adults (AUCR_adults_):

(12) $${\mathrm{AUCR}}_{\mathrm{pediatrics}}=\frac{1}{1-\frac{Z\;ontogeny}{W\;allometry\;}*\left(1-\frac{1}{{AUCR}_{adults}}\right)}$$

where Z_ontogeny_ and W_allometry_ are defined for each approach as follows:

Approach A: Both are equal to 1, which, rearranging the equation, results in AUCR_pediatrics_ = AUCR_adults_.

Approach B: Z_ontogeny_ equals the ontogeny functions (Equations (4)–(8)) and W_allometry_ equals 1, yielding:

$${\mathrm{AUCR}}_{\mathrm{pediatrics}}=\frac{1}{1-Z_{ontogeny}*\left(1-\frac{1}{{AUCR}_{adults}}\right)}$$

Approach C: Z_ontogeny_ equals the ontogeny functions (Equations (4)–(8)), and W_allometry_ equals the allometric correction (Equation (10)), using the full Equation (12).

An analogous static method was developed for describing the extent of CYP-mediated DDI in adults following induction of a metabolic pathway [22]:

(13) $$\frac{{AUC}^{*}}{AUC}\;=\frac{1}{1+CR*IC}$$

This methodology can therefore be extended to pediatric subjects similarly to the inhibition case:

(14) $${\mathrm{AUCR}}_{\mathrm{pediatrics}}=\frac{1}{1+\frac{Z\;ontogeny}{W\;allometry\;}*\left(\frac{1}{{AUCR}_{adults}}-1\right)}$$

The same definitions of Z_ontogeny_ and W_allometry_ for Approaches A-C, which are described above for the inhibition case, can be applied to the induction scenario.

### 4.2. Model Validation

The AUCR values reported by Salem et al. [3] were used for model verification based on visual inspection of plots comparing predicted *versus* observed AUCR values (Table 1). The observed pediatric AUCR values reported by Salem et al. [3] were derived from clinical DDI studies conducted in pediatric patients and reported in the literature. Unlike adult DDI studies, ethical constraints preclude the conduct of DDI studies in healthy pediatric volunteers; therefore, the observed values may reflect the influence of disease status and concomitant therapies on drug PK. For observed AUCR_pediatric_ involving patients of varying ages, the predicted AUCR_pediatric_ was calculated using the age corresponding to the midpoint of the reported age range. Consistent with other PK prediction models in both adult [24,25,26] and pediatric populations [27], predictions were considered accurate if at least 90% of the predicted AUCR values fell within the 50–200% of the corresponding observed AUCRs. Moreover, to assess the performance of the prediction approach, the predicted AUCR_pediatric_ values were compared with Guest’s intervals [23], which are frequently used in PK prediction studies.

## Figures and Tables

**Table 1:** Predicted AUCR_pediatric_ values obtained with the three approaches: translation from observed adult AUCR values (Approach A); approach including only an ontogeny correction factor (Approach B); and approach including both an ontogeny and an allometric correction factor (Approach C).

Victim Drug	Perpetrator Drug	Main CYP Involved	Age	Observed AUCR_pediatric_	Predicted AUCR		
					Approach C	Approach B	Approach A
Bupivacaine	Diazepam	CYP3A4	2–10	1.70	1.72	1.21	1.25
Carbamazepine	Erythromycin	CYP3A4	6	2.17	2.78	1.42	1.49
Carbamazepine	Erythromycin	CYP3A4	9	3.17	2.13	1.44	1.49
Carbamazepine	Erythromycin	CYP3A4	8	2.00	2.29	1.44	1.49
Carbamazepine	Erythromycin	CYP3A4	9	1.63	2.13	1.45	1.49
Carbamazepine	Valproate	CYP3A4	9	0.92	1.12	1.07	1.07
Carbamazepine	Valproate	CYP3A4	9	0.82	1.12	1.07	1.07
Chloroquine	Chlorpheniramine	CYP2C19	6–12	1.73	1.00	1.00	0.79
Cyclophosphamide	Fluconazole	CYP2C19	2 m–18 y	1.29	1.00	1.00	1.79
Cyclosporine A	Ketoconazole	CYP3A4	3	1.01	1.47	1.12	1.15
Cyclosporine A	Norfloxacin	CYP3A4	10	1.64	1.27	1.15	1.16
Digoxin	Amiodarone	CYP3A4	0.5–18	3.53	2.61	1.56	1.62
Digoxin	Carvedilol	CYP3A4	2 w–7.8 y	1.90	1.45	1.13	1.16
Efavirenz	Rifampicin	CYP2B6	3–15	0.97	0.99	1.00	0.97
Etopside	Cyclosporine A	CYP3A4	8 m–17 y	1.47	1.48	1.23	1.25
Etopside	Cyclosporine A	CYP3A4	4.4–20.7	1.90	1.36	1.24	1.25
Imipramine	Carbamazepine	CYP1A2	6–16	1.06	1.01	1.00	1.06
Lidocaine	Clonidine	CYP3A4	1–9	0.64	1.57	1.18	1.21
Phenytoin	Chloramphenicol	CYP2C9	10–108 m	0.94	1.61	1.19	1.60
Phenytoin	Co-trimoxazole	CYP2C9	4	1.27	1.73	1.19	1.52
Ritonavir	Efavirenz	CYP2B6	5.7–16.3	1.27	1.01	1.01	1.09
Theophylline	Terbutaline	CYP1A2	7–11	0.79	0.99	0.99	0.93
Theophylline	Salbutamol	CYP1A2	19 m	0.42	0.79	0.93	0.82
Theophylline	Salbutamol	CYP1A2	5–13	0.54	0.97	0.98	0.83
Theophylline	Phenobarbitone	CYP1A2	8.86	0.70	0.98	0.99	0.88

AUCR: AUC ratio; y: years; m: months; w: weeks; where not specified, age is reported in years. Main CYP involved: primary cytochrome P450 enzyme responsible for the metabolism of the victim drug.

**Table 2:** Performance analysis of the three approaches: translation from observed adult AUCR values (Approach A); approach including only an ontogeny correction factor (Approach B); and both an ontogeny and an allometric correction factor (Approach C).

Victim Drug	Perpetrator Drug	AUCR Predicted/AUCR Observed		
		Approach C	Approach B	Approach A
Bupivacaine	Diazepam	1.01	0.71	0.74
Carbamazepine	Erythromycin	1.28	0.65	0.69
Carbamazepine	Erythromycin	0.67	0.45	0.47
Carbamazepine	Erythromycin	1.15	0.72	0.75
Carbamazepine	Erythromycin	1.31	0.89	0.91
Carbamazepine	Valproate	1.22	1.16	1.16
Carbamazepine	Valproate	1.37	1.30	1.30
Chloroquine	Chlorpheniramine	0.55	0.57	0.46
Cyclophosphamide	Fluconazole	0.78	0.78	1.39
Cyclosporine A	Ketoconazole	1.46	1.11	1.14
Cyclosporine A	Norfloxacin	0.77	0.70	0.71
Digoxin	Amiodarone	0.74	0.44	0.46
Digoxin	Carvedilol	0.76	0.59	0.61
Efavirenz	Rifampicin	1.02	1.03	1.00
Etopside	Cyclosporine A	1.01	0.84	0.85
Etopside	Cyclosporine A	0.72	0.65	0.66
Imipramine	Carbamazepine	0.95	0.94	1.00
Lidocaine	Clonidine	2.45	1.84	1.89
Phenytoin	Chloramphenicol	1.71	1.27	1.70
Phenytoin	Co-trimoxazole	1.36	0.94	1.20
Ritonavir	Efavirenz	0.80	0.80	0.86
Theophylline	Terbutaline	1.25	1.25	1.18
Theophylline	Salbutamol	1.88	2.21	1.95
Theophylline	Salbutamol	1.80	1.81	1.54
Theophylline	Phenobarbitone	1.40	1.41	1.26

AUCR: AUC ratio.

## Data Availability

The original contributions presented in this study are included in the article. Further inquiries can be directed to the corresponding authors.
